# Sperm Competition, Sperm Numbers and Sperm Quality in Muroid Rodents

**DOI:** 10.1371/journal.pone.0018173

**Published:** 2011-03-25

**Authors:** Laura Gómez Montoto, Concepción Magaña, Maximiliano Tourmente, Juan Martín-Coello, Cristina Crespo, Juan José Luque-Larena, Montserrat Gomendio, Eduardo R. S. Roldan

**Affiliations:** 1 Reproductive Ecology and Biology Group, Museo Nacional de Ciencias Naturales (CSIC), Madrid, Spain; 2 Área de Zoología, Departamento de Ciencias Agroforestales, E.T.S. Ingenierías Agrarias, Universidad de Valladolid, Campus La Yutera, Palencia, Spain; University of Otago, New Zealand

## Abstract

Sperm competition favors increases in relative testes mass and production efficiency, and changes in sperm phenotype that result in faster swimming speeds. However, little is known about its effects on traits that contribute to determine the quality of a whole ejaculate (i.e., proportion of motile, viable, morphologically normal and acrosome intact sperm) and that are key determinants of fertilization success. Two competing hypotheses lead to alternative predictions: (a) sperm quantity and quality traits co-evolve under sperm competition because they play complementary roles in determining ejaculate's competitive ability, or (b) energetic constraints force trade-offs between traits depending on their relevance in providing a competitive advantage. We examined relationships between sperm competition levels, sperm quantity, and traits that determine ejaculate quality, in a comparative study of 18 rodent species using phylogenetically controlled analyses. Total sperm numbers were positively correlated to proportions of normal sperm, acrosome integrity and motile sperm; the latter three were also significantly related among themselves, suggesting no trade-offs between traits. In addition, testes mass corrected for body mass (i.e., relative testes mass), showed a strong association with sperm numbers, and positive significant associations with all sperm traits that determine ejaculate quality with the exception of live sperm. An “overall sperm quality” parameter obtained by principal component analysis (which explained 85% of the variance) was more strongly associated with relative testes mass than any individual quality trait. Overall sperm quality was as strongly associated with relative testes mass as sperm numbers. Thus, sperm quality traits improve under sperm competition in an integrated manner suggesting that a combination of all traits is what makes ejaculates more competitive. In evolutionary terms this implies that a complex network of genetic and developmental pathways underlying processes of sperm formation, maturation, transport in the female reproductive tract, and preparation for fertilization must all evolve in concert.

## Introduction

Sperm competition is a powerful selective force that has shaped male reproductive behavior, physiology, reproductive tract morphology, and gamete phenotype [1]–[3]. Competition between ejaculates takes place when two or more males mate with a female in any given receptive period and sperm from rival males compete to fertilize ova [4]. Sperm competition has been well documented in insects [2], [4], birds [5], [6], fishes [7], amphibians [8] and mammals [9].

A widespread response to an increase in levels of sperm competition is an increase in testes mass relative to body mass [2], [3], [9], [10]. Experimental studies have shown that there is a causal relationship between increased levels of sperm competition and larger relative testes mass [11], [12], and comparative analyses have shown that relative testes mass is closely associated with genetic paternity [13]. Thus, differences in relative testes mass among species are commonly used as a proxy for levels of sperm competition. Increases in relative testes mass often involve both an increase in the amount of sperm producing tissue and in the efficiency per unit of tissue [14]. This results in higher sperm numbers in sperm reserves, which translates into more sperm per ejaculate [15], [16]. Transfer of high sperm numbers at the time of copulation increases the chances of fertilization because of the considerable sperm losses along the female tract, with only a few sperm reaching the site of fertilization in mammals [17]. In competitive contexts, theoretical models suggest that males with more sperm should gain a greater share of paternity when mechanisms of sperm competition resemble a raffle [18], and experimental studies have shown that males which transfer more sperm per ejaculate gain more fertilizations (see reviews in [1]).

A great deal of interest has been placed also on the effect of sperm competition on sperm design (head shape and sperm dimensions) and sperm function (e.g., swimming velocity) in a wide variety of taxa because sperm design influences sperm swimming velocity which, in turn, would affect fertilization success [19]. Many studies have now shown a positive association between levels of sperm competition and the length of the sperm cell (reviewed in [20]), and although there have been contradictory results in comparative analyses among mammals [19]–[24], a recent study has shown a clear positive association also in this taxon [25]. It has been argued that sperm competition may also affect the shape of the sperm head [26] and evidence has been presented showing an association between levels of sperm competition and hook shape and size in the head of rodent spermatozoa [27]. Sperm swimming velocity has also received attention because the first spermatozoon that reaches the ovum will be more likely to engage in fertilization [28] and faster sperm seem to be advantageous in both non-competitive [29]–[31] and competitive contexts [32], [33]. Comparative studies have shown that there are direct associations between different descriptors of sperm swimming velocity and sperm competition levels [25], [34].

Whereas the role of sperm competition favoring increased sperm numbers (i.e., quantity) or changes in the sperm cell phenotype are well documented, less is known regarding the impact on other traits that collectively determine ejaculate quality and that are important determinants of fertilization success. Ejaculate quality traits traditionally include the proportion of motile and viable spermatozoa, the proportion of spermatozoa without abnormalities in the different sperm components, and spermatozoa with an intact acrosome, but no study has so far addressed comprehensive analyses of how they may be affected by sperm competition. These traits are important at different stages in the life of the sperm cell, are essential to overcome barriers in the female tract, and to undergo molecular and cellular changes needed to participate in fertilization [35]. Such traits are expected to influence an ejaculate's competitive ability. Thus, sperm motility is required to actively negotiate barriers in the female tract (i.e., cervix and utero-tubal junction in mammals), to swim along the oviduct towards the site of fertilization, and to vigorously penetrate the ovum vestments [36]. Adequate sperm motility depends on normal sperm morphology; abnormal spermatozoa are sometimes immotile or may move in an ineffective way, being incapable of reaching the ova [37] because they cannot negotiate the utero-tubal junction [38]. Acrosome integrity is critical when spermatozoa need to attach to the mucosa of the oviductal wall [39] and, later, when the spermatozoon reaches the ovum and has to penetrate the ovum vestments [40] and bind to and interact with the zona pellucida [35]. Finally, a considerable proportion of spermatozoa die during transit along the female reproductive tract, given all the challenges that they must face, and thus sperm survival (i.e., viability) is an important determinant of a male's reproductive success [41].

Few studies have examined the influence of sperm competition on these ejaculate quality traits, and most of them have analyzed a single trait in isolation. Sperm viability has been shown in insects to have an important influence on paternity at the intraspecific level [42] and it has been found to be higher in polyandrous than in monandrous insect species as an adaptation to sperm competition [43]. In a comparative study among primates, with data gathered from the literature, sperm motility appeared to be higher in multi-male than in single-male breeding systems, as well as showing a positive association with relative testes mass [15], although this study did not control for phylogenetic effects. Sperm motility has been shown to be important as a determinant of male reproductive success in the domestic fowl where, in competitive contexts (keeping the same number of sperm for each male), males showing better sperm motility sired the majority of offspring [32]. In mice, experiments manipulating the mating system have revealed that sperm competition promotes an increase in sperm motility on polyandrous mice lines when compared with monandrous ones [44]. In rodents, sperm competition also seems to increase the proportion of sperm which become ready to interact with the ovum and the sensitivity to ovum signals [45]. However, no study has so far addressed the study of different ejaculate quality traits in an integrated manner in a group of species that differ in levels of sperm competition.

It is not known if sperm competition differentially favors improvements of specific traits which may play a more relevant role in competitive contexts, or if all sperm quality traits co-evolve under this selective pressure because they play complementary roles in outcompeting rival ejaculates. There may be constraints to the co-evolution of different sperm quality traits since sperm are costly to produce [46]–[50]. Thus, traits which are expensive in terms of energy or time may be traded-off against other traits which do not confer major competitive advantages. Theoretical studies have predicted trade-offs between sperm numbers and size [4], [51]–[53], which have been supported by empirical evidence in cases of sperm gigantism [54], [55]. Other types of trade-offs include sperm swimming velocity versus longevity in the sea urchin [30], and a trade-off between sperm length and longevity in the Atlantic salmon [56] and across fish species [7]. However, other studies have not found evidence of trade-offs between sperm traits both within [31], [33], [56], [57] and between species [34].

The order Rodentia is the largest and most diverse mammalian order. Muroid rodents (families Muridae, Cricetidae and Arvicolidae) represent a group of phylogenetically close species that experience different levels of sperm competition [13] and thus show a considerable range of variation in testes mass [58], and also a wide range of sperm sizes [59] and sperm morphology [26]. Therefore, this group represents an ideal system to understand the impact of sperm competition on ejaculate traits and analyze the degree of co-variation between sperm quantity (numbers) and indicators of ejaculate quality.

In this study, we investigated the impact of sperm competition on sperm numbers, sperm morphology, acrosome integrity, sperm viability, and sperm motility in 18 muroid rodent species that differ widely in levels of sperm competition. To the best of our knowledge, this represents the first comparative study of ejaculate traits between closely related species in which fresh sperm samples were collected and analyzed under similar conditions, thus eliminating any other confounding factors which have been shown to limit the validity of comparative studies.

## Results

### Body Measures

**Figure 1:**
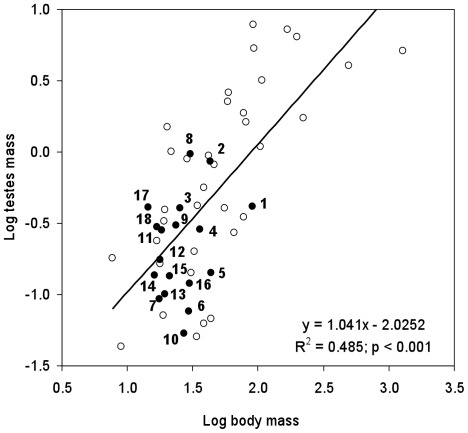
Relations between log body mass and log testes mass in 53 rodent species. Data points represent species values. Empty circles: muroid species from Kenagy and Trombulak [58] (N = 35). Filled circles: muroid species from the present study (N = 18). Numbers denote species as follows: 1, *Arvicola terrestris*; 2 *Chionomys nivalis*; 3, *Clethrionomys glareolus*; 4, *Microtus arvalis*; 5, *Microtus cabrerae*; 6, *Microtus duodecimcostatus*; 7, *Microtus lusitanicus*; 8, *Apodemus sylvaticus*; 9, *Mus cookii*; 10, *Mus famulus*; 11, *Mus macedonicus*; 12, *Mus musculus bactrianus*; 13, *Mus musculus castaneus*; 14, *Mus musculus domesticus*; 15, *Mus musculus musculus*; 16, *Mus pahari*; 17, *Mus spicilegus*; 18, *Mus spretus*.

In our study sample, body weights of muroid species varied from 14.49±0.49 g (mean ± SEM) in *Mus spicilegus*, the smallest species, to 91.56±4.47 g in *Arvicola terrestris*, the largest one (Table 1), showing a high coefficient of variation (CV) of 61.5%. Testes mass showed more variation (CV = 87.5%) with a range from 0.053±0.007 g in *Mus famulus* to 0.961±0.054 g in *Apodemus sylvaticus*, thus revealing a considerable level of variation among muroid species. Relative testes mass ranged from 0.134 in *Mus famulus* to 2.236 in *Apodemus sylvaticus* (CV = 78.4%); values for the 18 species in this study fell within the range exhibited by muroid rodents (Fig. 1) [58]. There was a ∼20-fold difference in relative testes mass between the species with the lowest and highest values, although there was only a ∼6-fold difference in body mass among these species. Thus, differences in testes mass vastly exceeded the differences in organ size that could be related to changes in body size because of the dilution effect of a larger body volume (reviewed in [9]).

**Table 1:** Body mass, testes mass and relative testes mass of muroid rodents.

Species	N	Body mass (g)	Testes mass (g)	Relative testes mass
*Arvicola terrestris*	5	91.56±4.47	0.411±0.027	0.409
*Chionomys nivalis*	6	43.64±1.35	0.851±0.061	1.499
*Clethrionomys glareolus*	6	25.65±1.47	0.401±0.048	1.063
*Microtus arvalis*	8	36.40±3.17	0.285±0.023	0.577
*Microtus cabrerae*	6	44.27±2.47	0.142±0.025	0.247
*Microtus duodecimcostatus*	6	29.76±1.36	0.076±0.013	0.179
*Microtus lusitanicus*	3	17.73±0.88	0.093±0.023	0.327
*Apodemus sylvaticus*	8	30.43±1.70	0.961±0.054	2.236
*Mus cookie*	4	23.67±0.81	0.305±0.032	0.861
*Mus famulus*	3	27.40±0.77	0.053±0.007	0.134
*Mus macedonicus*	3	18.40±0.67	0.282±0.022	0.966
*Mus musculus bactrianus*	3	18.06±3.04	0.175±0.008	0.609
*Mus musculus castaneus*	3	19.50±0.33	0.100±0.006	0.326
*Mus musculus domesticus*	4	16.36±0.97	0.135±0.005	0.506
*Mus musculus musculus*	5	21.13±1.06	0.134±0.003	0.411
*Mus pahari*	5	30.08±0.33	0.118±0.007	0.277
*Mus spicilegus*	5	14.49±0.49	0.409±0.012	1.682
*Mus spretus*	5	17.01±0.48	0.295±0.008	1.072

Values are mean ± SEM. Relative testes mass was calculated as the ratio of observed testes mass to the predicted testes mass Y. Predicted testes mass Y for each species was calculated following Kenagy and Trombulak's [58] formula for rodents: Y = 0.031X^0.77^, where X is the observed body mass.

### Sperm Traits

Total sperm numbers in epididymides (i.e., sperm reserves) varied considerably, from 4.5±1.2×10^6^ spermatozoa to 129.4±31.5×10^6^ spermatozoa (Table 2). Coefficient of variation of total sperm number across species was very high (CV = 75.5%).

**Table 2:** Sperm numbers and quality in muroid rodents.

Species	N	Total sperm number (×10^6^)	% normal sperm	% acrosome integrity	% live sperm	% motile sperm
*Arvicola terrestris*	5	36.5±6.8	88.1±3.8	95.9±1.4	68.3±6.9	67.0±3.0
*Chionomys nivalis*	6	129.4±31.5	87.8±1.8	96.0±1.0	74.3±4.5	88.3±1.1
*Clethrionomys glareolus*	6	43.2±10.7	90.1±1.4	91.9±3.5	69.7±6.8	78.3±3.3
*Microtus arvalis*	8	41.6±7.1	90.2±0.8	98.1±0.3	66.6±6.1	86.9±1.9
*Microtus cabrerae*	6	7.6±0.9	76.5±3.4	96.1±1.2	51.2±7.6	55.8±6.5
*Microtus duodecimcostatus*	6	4.5±1.2	69.4±6.7	47.3±7.6	74.9±4.8	45.0±1.8
*Microtus lusitanicus*	3	27.7±14.0	81.3±6.9	86.8±5.4	75.2±5.1	78.3±1.7
*Apodemus sylvaticus*	8	110.1±12.0	93.4±1.6	96.1±1.1	74.8±3.2	82.5±2.8
*Mus cookii*	4	62.2±9.9	87.5±2.6	71.0±3.6	75.8±2.6	90.8±7.0
*Mus famulus*	3	44.9±0.7	74.0±3.0	65.3±5.7	68.3±3.4	41.7±11.7
*Mus macedonicus*	3	69.9±12.5	79.0±3.6	86.3±3.2	72.7±0.9	70.0±5.8
*Mus musculus bactrianus*	3	43.6±8.5	84.7±3.7	67.7±2.9	70.3±2.7	40.0±10.0
*Mus musculus castaneus*	3	26.5±16.3	85.0±4.5	72.0±2.6	63.3±6.5	63.3±8.8
*Mus musculus domesticus*	4	18.4±5.1	80.3±0.8	68.8±3.8	60.3±3.9	87.5±2.5
*Mus musculus musculus*	5	23.2±2.0	73.0±3.2	70.0±3.0	88.8±0.6	71.7±0.7
*Mus pahari*	5	9.1±0.3	75.6±0.6	73.0±2.4	70.8±0.6	50.0±0.02
*Mus spicilegus*	5	99.4±15.8	84.5±2.9	77.0±3.3	93.8±1.1	90.5±1.4
*Mus spretus*	5	48.0±3.4	79.5±2.5	70.8±2.6	97.3±0.3	98.3±0.3

Values are mean ± SEM.

Values of percentage of normal sperm ranged between 69.4±6.7% and 93.4±1.6% (Table 2). This was the sperm quality trait with less variation (CV = 8.2%). The percentage of acrosome integrity (percentage of spermatozoa with intact acrosomes) showed a low value of CV (18.1%) and it ranged from 47.3±7.6% to 98.1±0.3% (Table 2). Percentage of live sperm also exhibited a low range of variation (CV = 15.3%) with a range from 51.2±7.6% to 97.3±0.3% (Table 2). Finally, percentage of motile sperm was the sperm quality trait with more variation, showing the highest coefficient of variation (25.9%) and ranging from 40±10% to 98.3±0.3% (Table 2).

On the whole, species with higher values of relative testes mass turned out to be the species with higher total sperm number and higher sperm quality (Table 2). *Apodemus sylvaticus*, the species with the highest level of sperm competition (as suggested by the high relative testes mass) was also the species with the best sperm quality, and one of the species with higher sperm numbers, whereas *Microtus duodecimcostatus*, with very low level of sperm competition (as indicated by a low relative testes mass), showed the lowest values of sperm numbers and quality.

### Correlations Between Sperm Traits and Cluster Analysis

**Figure 2:**
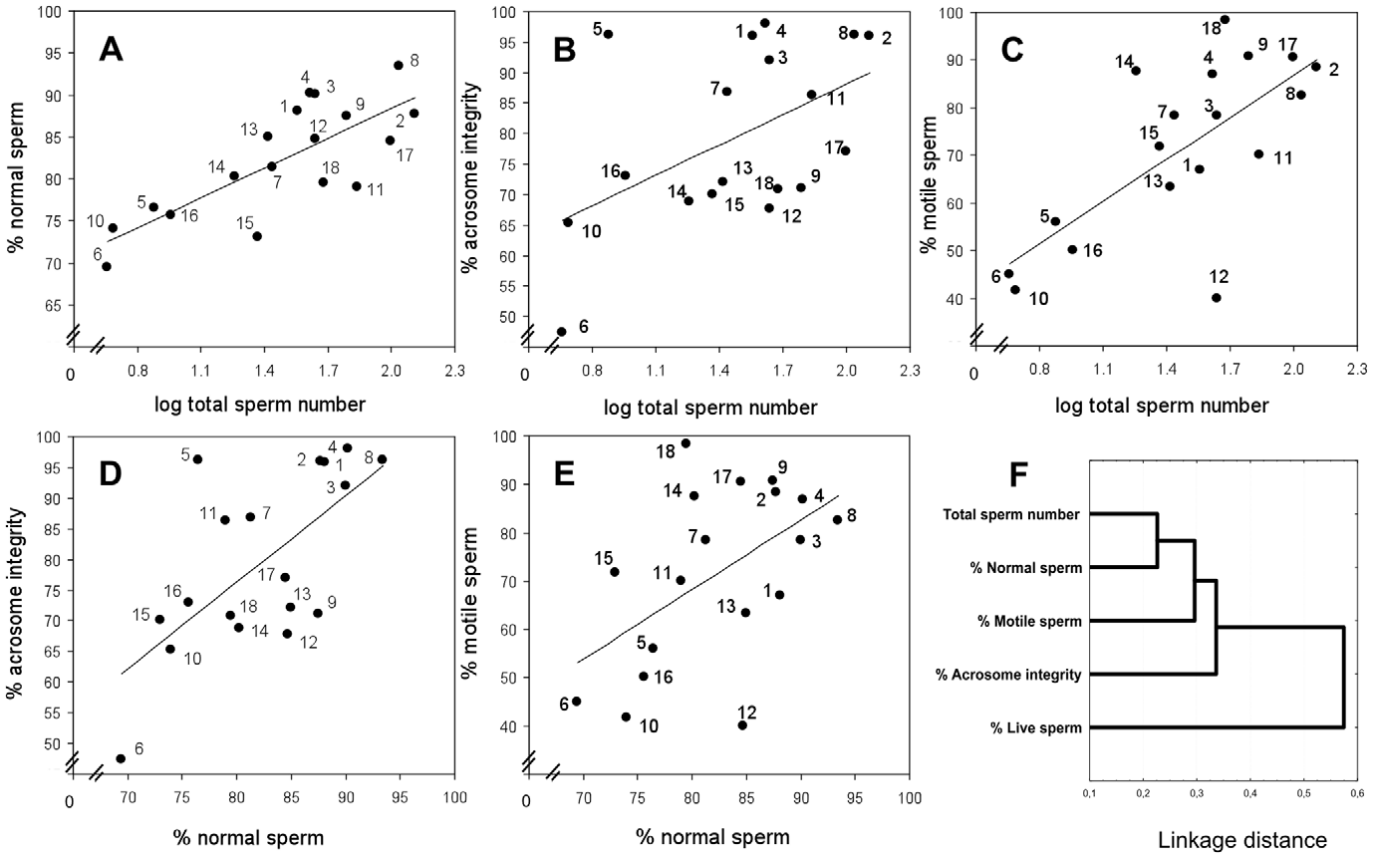
Associations between sperm parameters. Association between total sperm number and (A) % normal sperm, (B) % acrosome integrity and (C) % motile sperm. Association between % normal sperm and (D) % acrosome integrity and (E) % motile sperm. Numbers in each panel denote species as follows: 1, *Arvicola terrestris*; 2 *Chionomys nivalis*; 3, *Clethrionomys glareolus*; 4, *Microtus arvalis*; 5, *Microtus cabrerae*; 6, *Microtus duodecimcostatus*; 7, *Microtus lusitanicus*; 8, *Apodemus sylvaticus*; 9, *Mus cookii*; 10, *Mus famulus*; 11, *Mus macedonicus*; 12, *Mus musculus bactrianus*; 13, *Mus musculus castaneus*; 14, *Mus musculus domesticus*; 15, *Mus musculus musculus*; 16, *Mus pahari*; 17, *Mus spicilegus*; 18, *Mus spretus*. (F) Cluster diagram showing relations between sperm parameters based on the raw correlation matrix with single linkage joining rule (distance metric = 1−*r*) (see correlation matrix in Table 3).

In phylogenetically-controlled analyses, total sperm number was positively correlated to percentage of normal sperm (*P*<0.0001) (Table 3, Fig. 2A), percentage of acrosome integrity (*P*<0.05) (Table 3, Fig. 2B) and percentage of motile sperm (*P*<0.001) (Table 3, Fig. 2C). Moreover, percentage of normal sperm presented positive correlations with percentage of acrosome integrity (*P*<0.001) (Table 3, Fig. 2D) and percentage of motile sperm (*P*<0.01) (Table 3, Fig. 2E). Percentage of live sperm did not correlate to total sperm number or to any sperm quality trait (Table 3).

**Table 3:** Correlation matrix of sperm traits analyzed.

	Total sperm number	% normal sperm	% acrosome integrity	% live sperm	% motile sperm
Total sperm number		**0.837**	**0.577**	0.400	**0.787**
% normal sperm	****		**0.722**	0.079	**0.661**
% acrosome integrity	*	***		0.144	0.428
% live sperm	-	-	-		0.463
% motile sperm	***	**	-	-	

Bold numbers indicate significant effect sizes *r* with *P*<0.05. Symbols:

*P<0.05;

**P<0.01;

***P<0.001;

****P<0.0001.

A cluster diagram (Fig. 2F), based on effect size *r* values, was obtained from the correlation matrix (Table 3) and it provided a visual representation of how sperm traits were associated. Total sperm number and percentage of normal sperm showed the strongest association between sperm traits and formed a cluster clearly related to percentage of motile sperm. Percentage of acrosome integrity was closely associated with the cluster formed by total sperm number, percentage of normal and motile sperm. Finally, percentage of live sperm was the trait that was related the least to the other sperm traits.

### Multiple Regression and GLS Analyses

**Figure 3:**
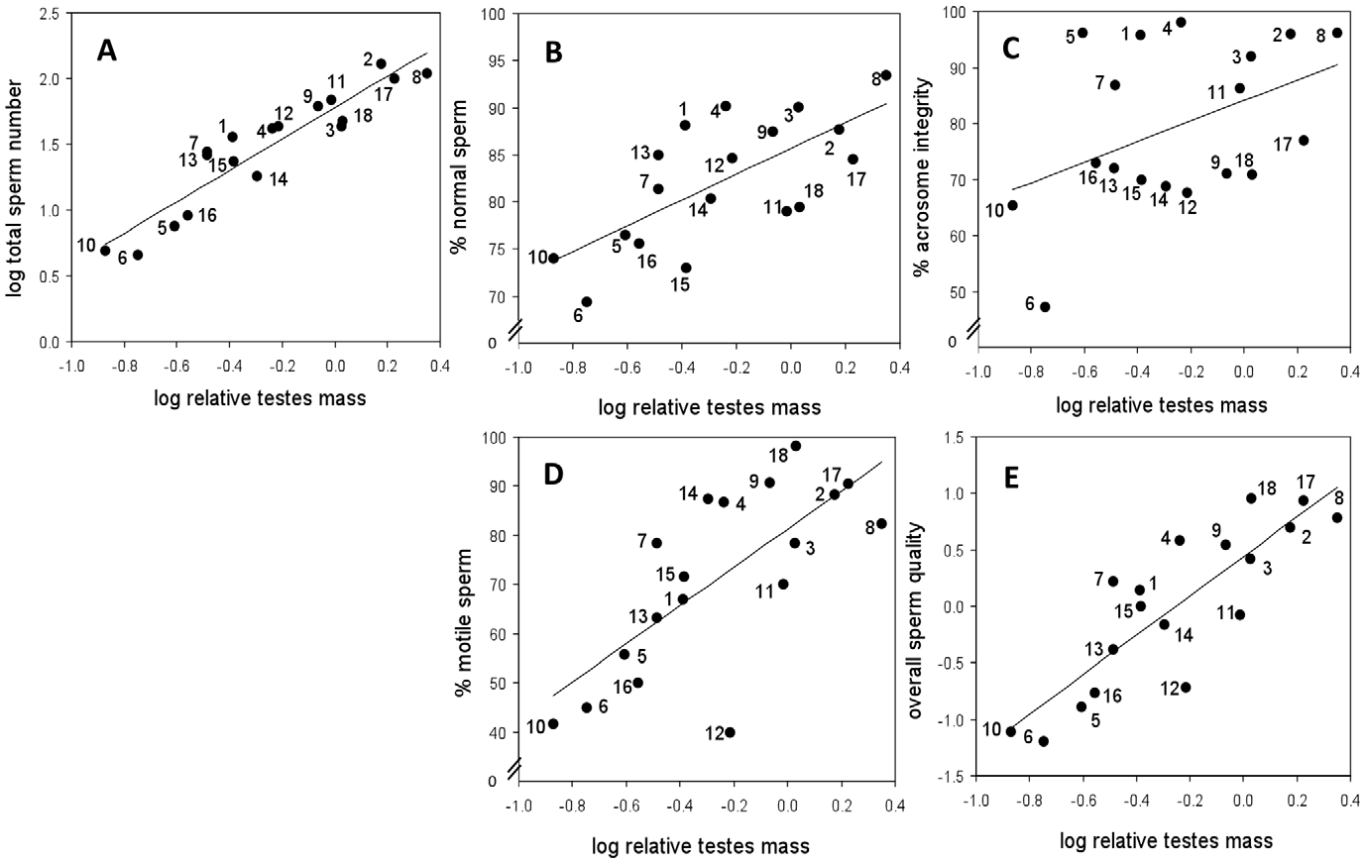
Relations between relative testes mass and sperm quantity and quality traits. (A) total sperm number, (B) % normal sperm, (C) % acrosome integrity, (D) % motile sperm and (E) overall sperm quality. N = 18 muroid species. Numbers in each panel denote species as in Fig. 2. See results of multiple regression analyses in Table 4.

In phylogenetically-controlled analyses, we found positive associations between testes mass corrected for body mass (thereafter, relative testes mass) and total sperm number (*P*<0.0001) (Table 4, Fig. 3A), percentage of normal sperm (*P*<0.0001) (Table 4, Fig. 3B), percentage of acrosome integrity (*P*<0.01) (Table 4, Fig. 3C), and percentage of motile sperm (*P*<0.001) (Table 4, Fig. 3D). On the other hand, percentage of live sperm was not related to relative testes mass (Table 4).

**Table 4:** Phylogenetically-controlled multiple regression analyses of sperm numbers and sperm quality traits in relation to body mass and testes mass.

Dependent variable	Predictor	Adjusted *R* ^2^	Slope	*F*	*P*	Lambda (λ)	Effect size(r)	Effect size CLs
Total sperm number	Body mass	0.866	−0.60	0.07	0.7951	<0.0001^†,^*	0.07	−0.49 to 0.63
	Testes mass		1.16	111.75	**<1×10^−7^**		0.94	**1.16 to 2.29**
% normal sperm	Body mass	0.699	0.29	3.69	0.0738	<0.0001^†,^*	0.44	−0.08 to 1.04
	Testes mass		16.13	37.84	**<0.0001**		0.85	**0.68 to 1.81**
% acrosome integrity	Body mass	0.571	22.54	11.39	**0.0042**	<0.0001^†,^*	0.66	**0.22 to 1.35**
	Testes mass		22.66	13.24	**0.0024**		0.68	**0.27 to 1.40**
% live sperm	Body mass	0.206	−22.04	3.71	0.0733	<0.0001^†,^*	0.44	−0.09 to 1.04
	Testes mass		9.45	2.71	0.1202		0.39	−0.15 to 0.97
% motile sperm	Body mass	0.546	−31.93	1.48	0.2425	<0.0001^†,^*	0.30	−0.26 to 0.87
	Testes mass		35.51	21.00	**0.0003**		0.76	**0.44 to 1.57**
Overall sperm quality	Body mass	0.747	−0.96	0.19	0.6678	<0.0001^†,^*	0.11	−0.45 to 0.68
	Testes mass		1.67	52.09	**<1×10^−5^**		0.88	**0.81 to 1.95**

All tests were conducted with 15 df. The superscripts following λ value indicate significance levels (^†^ n.s.; *P<0.05) in likelihood ratio tests against models with λ = 0 (first position) and λ = 1 (second position). The effect size *r* was calculated from the *F* values and we present its noncentral 95% confidence limits (CLs) too. Confidence intervals excluding 0 indicate statistically significant relationships. *P* values and CLs that indicate statistical significance are shown in bold.

When all the traits that determine ejaculate quality (percentage of normal sperm, percentage of acrosome integrity, percentage of live sperm and percentage of motile sperm) were analyzed together in a PCA, two factor scores (1 and 2) explained 85% of the variance (Table 5). This “overall sperm quality” trait was very strongly associated (*P*<0.0001) to relative testes mass (Table 4, Fig. 3E).

**Table 5:** Factor loadings of the four sperm quality traits obtained by means of a principal component analysis.

	Factor loadings	
Sperm quality traits	Factor score 1	Factor score 2
% normal sperm	0.899	0.069
% acrosome integrity	0.892	−0.192
% live sperm	−0.169	0.938
% motile sperm	0.626	0.680
eigenvalue	2.062	1.348
variance explained (%)	51.5	33.7

Factors were rotated by Varimax rotation method.

## Discussion

Despite being phylogenetically very close, muroid species examined in this study showed high variability of testes masses, despite much lower levels of variation in body mass. Consequently, this group represents an ideal model to study the effects of sperm competition since it shows a wide range of variation in relative testes mass that is indicative of considerable differences in levels of sperm competition among these species. Major differences were also found in terms of both sperm numbers and traits that determine ejaculate quality which were associated with levels of sperm competition in phylogenetically-controlled analyses. In addition, all sperm traits examined (quantity and quality) seem to co-evolve and no evidence suggestive of any kind of trade-offs was found. Thus, our findings provide robust evidence that sperm competition favors an increase in both sperm numbers, as well as the proportion of normal, motile and acrosome intact sperm. The only trait which followed a different pattern was sperm viability (i.e., percentage of live sperm) which was unrelated to other sperm quality traits and did not respond to increased levels of sperm competition.

Two competing hypotheses could explain the evolutionary response of sperm quantity and quality traits under increased levels of sperm competition. Sperm traits would be expected to co-evolve if all of them play complementary roles which jointly determine fertilization success and competitive ability. In contrast, sperm traits may follow different evolutionary trends if there are budget constraints because improvements on all traits are too costly to afford [46], [47] and traits which play a greater role in enhancing ejaculate competitiveness are traded-off against less relevant traits. Earlier comparative studies, based on data compiled from the literature, did not find evidence for positive co-variance of sperm numbers and sperm motility in primates [15]. In an intraspecific analysis of semen traits of red deer natural populations [31] no trade-offs were observed, which suggested co-evolution to maximize fertilizing efficiency. In the present interspecific study no negative associations suggestive of trade-offs were observed and the majority of sperm traits (with the exception of live spermatozoa) co-varied positively. Thus, total sperm number was strongly associated with the percentage of normal sperm, which in turn was closely related to percentage of motile sperm. Finally, this cluster was closely associated with percentage of acrosome integrity. In contrast, percentage of live sperm seemed unrelated to the other sperm traits.

Evolutionary improvements in both sperm numbers and ejaculate quality traits are driven by sperm competition since, in phylogenetically-controlled analyses, all sperm traits (except live sperm) were associated with differences between species in relative testes mass. Thus, sperm competition not only has an important effect on sperm production, as revealed in previous studies [1]–[3], [9], but it also selects for an improvement in different traits that determine the quality of the ejaculate.

The fertilization process involves many steps all of which must be succesfully overcome by ejaculates in order to fertilize. Ejaculates need to contain enough sperm with normal morphology, since abnormal sperm cannot participate in fertilization [60]–[62], and males with a low proportion of normal sperm in the ejaculate suffer low fertility [31]. A high proportion of abnormal spermatozoa may be caused by genetic factors [63]–[66], and several null mutations are known to cause abnormal sperm morphology in mice [66]. Sperm morphology also influences sperm motility, and for ejaculates to be able to achieve fertilization they require a considerable proportion of sperm with vigorous motility which is needed to travel along the female tract, overcome several physical barriers, and penetrate ovum vestments [32]. Variation among species in the proportion of motile sperm was high, which agrees with previous studies which have found high variation in this trait among field-trapped rodents [67], possibly because sperm motility is strongly influenced by environmental factors [68]. In addition, an intact acrosome is needed for sperm to interact with the female tract and ova [40], so a certain proportion of sperm need to retain acrosome integrity for fertilization to occur. It has also been suggested that the acrosome is used by sperm to attach to each other forming trains which presumably swim faster than single sperm [69]. All these ejaculate traits have been shown to influence male fertilization success [31], [66], [70], and our study suggests that when there is competition against rival sperm sexual selection favors improvements in all of them. In contrast, the proportion of live spermatozoa seems to be unaffected by sperm competition in muroid rodents, contrary to evidence found in insects [42], [43] and appears not to be related to any other sperm trait [71]. Its low variation could be explained because intense selection on this trait due to its importance in determining reproductive success [9] may result in uniformly high values [31]. Alternatively this result could be related to the fact this trait is particularly prone to be affected by the hostility of the female tract, so a more precise test of levels of sperm mortality would need to evaluate sperm viability well after sperm have faced a number of challenges in the female tract.

An “overall sperm quality” parameter obtained by principal component analysis was positively and very strongly associated to relative testes mass. Use of principal component analysis to reduce several variables to few factors that may encapsulate information on several reproductive traits such as sperm quality has proven useful before [31], [72]. This novel result highlights the fact that an overall measure that integrates different quality traits is more strongly associated with levels of sperm competition than any single trait analyzed separately. This finding strongly suggests that different sperm quality traits contribute in complementary ways to maximize fertilization efficiency and competitive ability and, as a consequence, they evolve in concert in response to increased levels of sperm competition.

The results of this study have important evolutionary implications because they suggest that the joint effects of sperm competition on so many different sperm quantity and quality traits is the result of very complex changes in terms of sperm formation and maturation, and the reproductive genes that control such processes. While an increase in sperm numbers is the result of an increase in testes size and production efficiency, the joint improvements in ejaculate quality traits involve changes at many different stages during spermatogenesis, as well as during maturational processes in both the male and female reproductive tracts. Thus, while the acrosome is formed during late stages of spermatogenesis and its morphology is modified in the epididymis, sperm acquire the ability to activate and express motility along transit in the epididymis [35]. In addition, traits such as sperm motility and the damage or loss of the acrosome are the result of the interation between sperm and the environment encountered in the female tract [36], [41]. Finally, traits such as sperm morphology seem to be under strong genetic control, since high levels of inbreeding cause an increase in sperm abnormalities [20], [64]. We conclude that the power of sperm competition to improve in an integrated manner an array of sperm quantity and quality traits is the result of changes in a complex network of genetic and developmental processes.

## Materials and Methods

### Animals

Adult males from eighteen species of muroid rodents were studied: *Arvicola terrestris*, *Chionomys nivalis*, *Clethrionomys ( = Myodes) glareolus*, *Microtus arvalis*, *Microtus cabrerae*, *Microtus duodecimcostatus*, *Microtus lusitanicus*, and *Apodemus sylvaticus* were trapped in the field during the breeding season; *Mus cookii*, *Mus famulus*, *Mus macedonicus*, *Mus musculus bactrianus*, *Mus m. castaneus*, *Mus m. domesticus*, *Mus m. musculus*, *Mus pahari*, *Mus spicilegus* and *Mus spretus* come from wild-derived colonies which have been kept in captivity for only a few generations and were purchased from the Institut des Sciences de l'Evolution, CNRS-Université Montpellier 2, France. Localities of origin for the different species are shown in Figure S1. Sample size varied between 3 and 8 individuals for each species (N = 4.9±0.4, mean ± SEM).

Males were kept in our animal facilities in individual cages, under standard laboratory conditions in environmentally-controlled rooms (20–24°C) on a 14 h light–10 h darkness photoperiod and provided with food and water *ad libitum*. All animal handling was done following Spanish Animal Protection Regulation RD1201/2005, which conforms to European Union Regulation 2003/65.

### Sperm Collection and Evaluation

Animals were sacrificed by cervical dislocation and were immediately weighed and dissected. Testes were removed and weighed. To obtain mature sperm, both epididymides and vasa deferentia were removed. Spermatozoa contained in each vas deferens were drawn to the epididymis by using a pair of forceps. Then, vasa deferentia were cut off and epididymides were placed in a 35 mm plastic culture dish containing Hepes-buffered modified Tyrode's medium (mT-H) under air [73]. The dishes were kept at 37°C on a warm plate during the whole procedure. The composition of mT-H medium was: 131.89 mM NaCl, 2.68 mM KCl, 0.49 mM MgCl_2_·6H_2_O, 1.80 mM CaCl_2_·2H_2_O, 0.36 mM NaH_2_PO_4_·2H_2_O, 5.56 mM glucose, 20 mM Hepes, 5 µg phenol red/ml, 50 µg kanamycin/ml, and 4 mg bovine serum albumin/ml (fraction V). Its pH was ∼7.55 at 20°C after adjusting it with NaOH and its osmolality was ∼295 mOsm/kg [73].

Incisions were made to the epididymides and these were incubated for 10 min to allow spermatozoa to swim out. The epididymides were discarded and the resulting sperm suspension was used for assessments. Because epididymides size varied among species, we used between 0.25 ml and 3 ml of mT-H medium and volume was recorded for the calculation of total sperm numbers.

Traits assessed were: total sperm number in both epididymides (“total sperm number”), percentage of normal spermatozoa (“% normal sperm”), percentage of spermatozoa with intact acrosomes (“% acrosome integrity”), percentage of live spermatozoa (“% live sperm”) and percentage of motile spermatozoa (“% motile sperm”).

Sperm motility was evaluated by placing 10 µl of the sperm suspension between a pre-warmed slide and a 22 mm×22 mm coverslip, and examining it at 100× magnification under phase-contrast optics. The percentage of motile sperm (ranging between 0%, when no motile spermatozoa were observed, and 100%, when all spermatozoa were moving) was estimated subjectively by at least two independent, experienced observers; estimations from the different observers were averaged and rounded to the nearest 5% value.

For the estimation of sperm concentration, an aliquot of the sperm suspension was fixed in 0.9% NaCl, 0.1% formaldehyde, 0.1% polyethylene glycol compound, and 2 mM EDTA pH 7.0 and then counted using a modified Neubauer chamber using phase contrast optics at 100× or 400× magnification. Total sperm number was calculated as follows: sperm concentration×volume of the sperm suspension.

Sperm morphology, viability and acrosome integrity were assessed in sperm smears stained first with eosin-nigrosin and subsequently with Giemsa [74]. Briefly, 5 µl sperm suspension and 10 µl eosin-nigrosin solution were mixed on a glass slide placed on a stage at 37°C and 30 s later the mix was smeared and allowed to air-dry. Smears were stained with Giemsa solution and mounted with DPX. Spermatozoa from *Microtus duodecimcostatus* did not stain well with eosin-nigrosin-Giemsa; thus sperm viability was assessed in smears stained only with eosin-nigrosin and acrosome integrity was assessed in smears stained with Coomassie blue as described for mouse sperm [75]. Smears were examined at 1000× under bright field and 200 spermatozoa per male were examined to evaluate sperm viability, morphology, and integrity of the acrosome.

Live spermatozoa were those excluding eosin (from the eosin-nigrosin stain). We quantified morphological abnormalities of the head, midpiece and principal plus terminal piece. Percentage of normal sperm was calculated as the proportion of spermatozoa with no morphological abnormalities out of all spermatozoa examined. Spermatozoa were grouped in three categories according to their acrosomal status: intact, damaged or lost [76]; only percentages of acrosome integrity for each male are reported.

### Statistical Analysis

In order to compare variability of body measures and sperm traits across the species, coefficients of variation (CV) were calculated as follows: CV = (SD * 100)/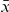, where SD = standard deviation, and 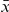 = mean. Variables were transformed to attain normal distributions. Normal distribution was tested by using a Kolmogorov–Smirnov normality test.

To explore relationships between total sperm number, % normal sperm, % acrosome integrity, % live sperm and % motile sperm, we calculated the effect size *r* of the correlations between the variables with phylogenetic correction. The level of test significance was adjusted to *P*<0.05. Using correlation matrix values between sperm numbers and sperm quality traits, we constructed a cluster diagram with single linkage-joining rule (distance metric = 1−*r*) to identify relationships between sperm traits.

A global measure of sperm quality (“overall sperm quality”) was obtained by means of a principal component analysis (PCA) to reduce potentially correlated variables of sperm quality (% normal sperm, % acrosome integrity, % live sperm, % motile sperm) to a single variable that would summarize the original information. This analysis extracted the first and second eigenvectors that summarized multivariate quality variation and best represented “quality components” [72].

To test whether different levels of sperm competition were associated with sperm numbers and quality across species, multiple regression analysis were performed using as dependent variables: total sperm number, % normal sperm, % acrosome integrity, % live sperm, % motile sperm and the global measure of sperm quality (“overall sperm quality”) calculated by PCA. Body mass and testes mass were used as predictor variables. Since predictor variables were related to each other (thus non orthogonal), they were added to the multiple regression analysis in the following order: body mass, testes mass, using a sequential (Type I) sum of squares.

As species may share character values as a result of a common ancestry rather than independent evolution [77], we used a generalized least-squares (GLS) approach in a phylogenetic framework [78] to control for phylogenetic effect on the associations of the variables. This method estimates a phylogenetic scaling parameter lambda (λ), which represents the transformation that makes the data fit the Brownian motion evolutionary model. When λ values are close to 0, variables are likely to have evolved independently of phylogeny, whereas λ values close to 1 indicate that the variables are strongly phylogenetically associated. GLS method allows for a variable degree of phylogenetic correction according to each tested model, accounting for different levels of phylogenetic association between different traits. The estimation of λ values and GLS analyses were performed using a code written by R. Freckleton for the statistical package R v.2.10.1 (R Foundation for Statistical Computing 2010) and the maximum likelihood value of λ was compared against the models with λ = 0 and λ = 1.

We reconstructed a phylogenetic tree of the species used in this study (Fig. S2) from partial phylogenies from the literature that were based on several mitochondrial, nuclear and ribosomal genes [79]–[89]. We also used *cytochrome b* sequences to clarify relationships among *Mus musculus* subspecies that were not resolved in previous studies. GenBank accession numbers for the sequences used are: *Arvicola terrestris*, AF159400; *Chionomys nivalis*, AY513848; *Clethrionomys glareolus*, AY309421; *Microtus arvalis*, AY220789; *Microtus cabrerae*, AY513788; *Microtus duodecimcostatus*, AY513796; *Microtus lusitanicus*, AY513812; *Apodemus sylvaticus*, AB033695; *Mus cookii*, AY057813; *Mus famulus*, AJ698872; *Mus macedonicus*, AY057808; *Mus musculus bactrianus*, HQ148567; *Mus m. castaneus*, AY057805; *Mus m. domesticus*, AY057807; *Mus m. musculus*, AY057804; *Mus pahari*, AY057814; *Mus spicilegus*, AY057809; *Mus spretus*, AY057810.

All statistical analyses were conducted with R v.2.10.1 and STATISTICA v.6.0, and *P* values were considered statistically significant at α<0.05.

In order to plot relative testes mass in the figures we calculated relative testes mass for each species following Kenagy and Trombulak's [58] formula for rodents: Y = 0.031X^0.77^, where Y is predicted testes mass in grams for the observed body mass X. Relative testes mass is calculated as the ratio of observed testes mass to the predicted testes mass Y. Relative testes mass was not used in any of the statistical analyses because this measure does not properly account for the allometric relationships between the variables [90].

## Supporting Information

Figure S1 **Species collection localities.** Numbers indicate species as follows: 1, *Arvicola terrestris* (Spain); 2, *Chionomys nivalis* (Spain); 3, *Clethrionomys glareolus* (Spain); 4, *Microtus arvalis* (Spain); 5, *Microtus cabrerae* (Spain); 6, *Microtus duodecimcostatus* (Spain); 7, *Microtus lusitanicus* (Spain); 8, *Apodemus sylvaticus* (Spain); 9, *Mus cookii* (Thailand); 10, *Mus famulus* (India); 11, *Mus macedonicus* (Bulgaria); 12 *Mus musculus bactrianus* (Iran); 13, *Mus musculus castaneus* (India); 14, *Mus musculus domesticus* (Morocco); 15, *Mus musculus musculus* (Georgia); 16, *Mus pahari* (Thailand); 17, *Mus spicilegus* (Ucrania); 18, *Mus spretus* (Morocco).
(TIF)

Figure S2 **Reconstructed phylogenetic tree of the muroid species used in this study.** The tree was constructed based on the literature and on the analysis of *cytochrome b* sequences Details are given in the Materials and Methods section.
(TIF)
