# Supplementary figures and images for: Sperm Competition, Sperm Numbers and Sperm Quality in Muroid Rodents
Source: PLoS One. 2011 Mar 25;6(3):e18173. doi: 10.1371/journal.pone.0018173 (PMC3064651; doi:10.1371/journal.pone.0018173)

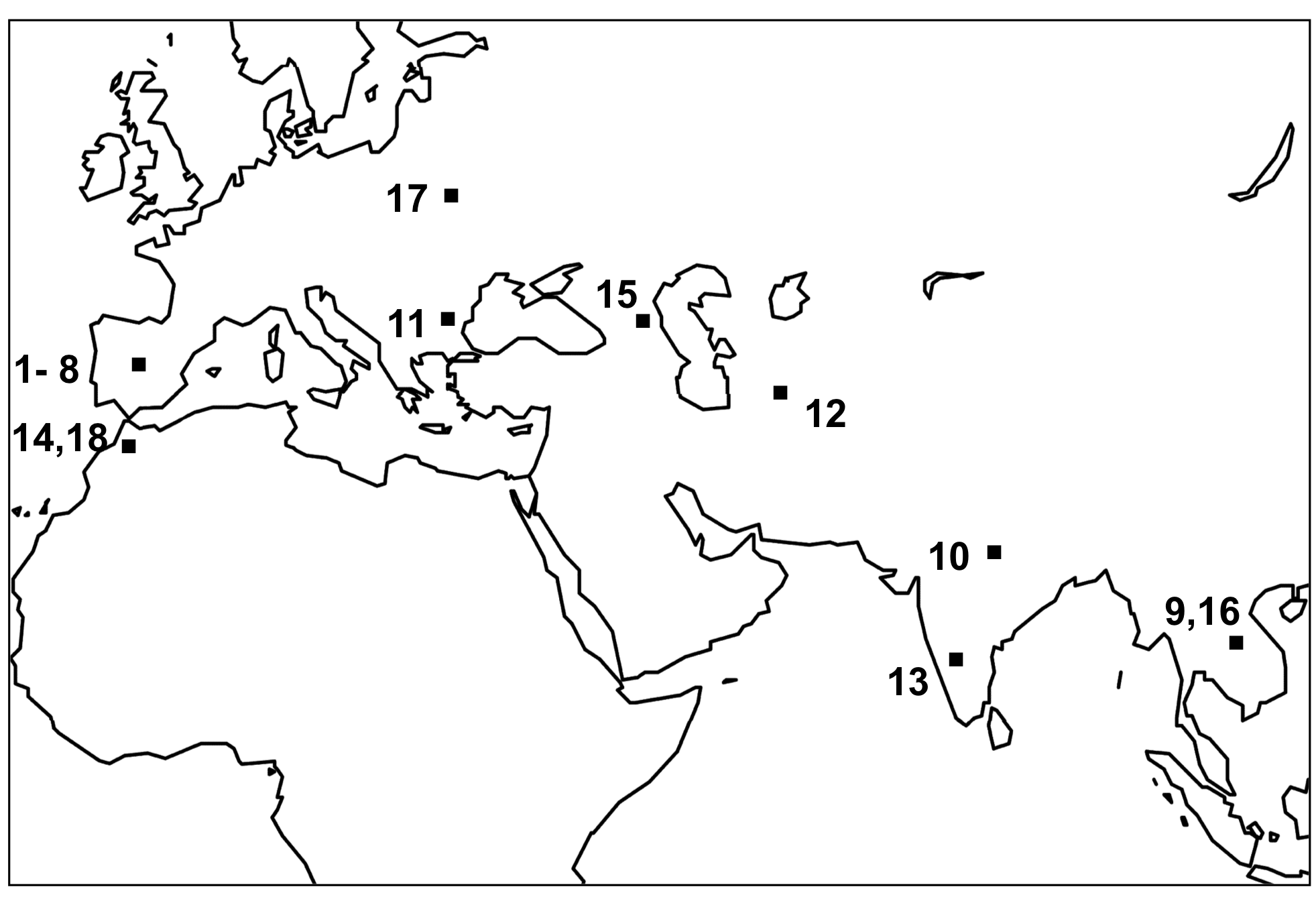

Supplement: Figure S1 — Species collection localities. Numbers indicate species as follows: 1, Arvicola terrestris (Spain); 2, Chionomys nivalis (Spain); 3, Clethrionomys glareolus (Spain); 4, Microtus arvalis (Spain); 5, Microtus cabrerae (Spain); 6, Microtus duodecimcostatus (Spain); 7, Microtus lusitanicus (Spain); 8, Apodemus sylvaticus (Spain); 9, Mus cookii (Thailand); 10, Mus famulus (India); 11, Mus macedonicus (Bulgaria); 12 Mus musculus bactrianus (Iran); 13, Mus musculus castaneus (India); 14, Mus musculus domesticus (Morocco); 15, Mus musculus musculus (Georgia); 16, Mus pahari (Thailand); 17, Mus spicilegus (Ucrania); 18, Mus spretus (Morocco). (TIF) [file pone.0018173.s001.tif]

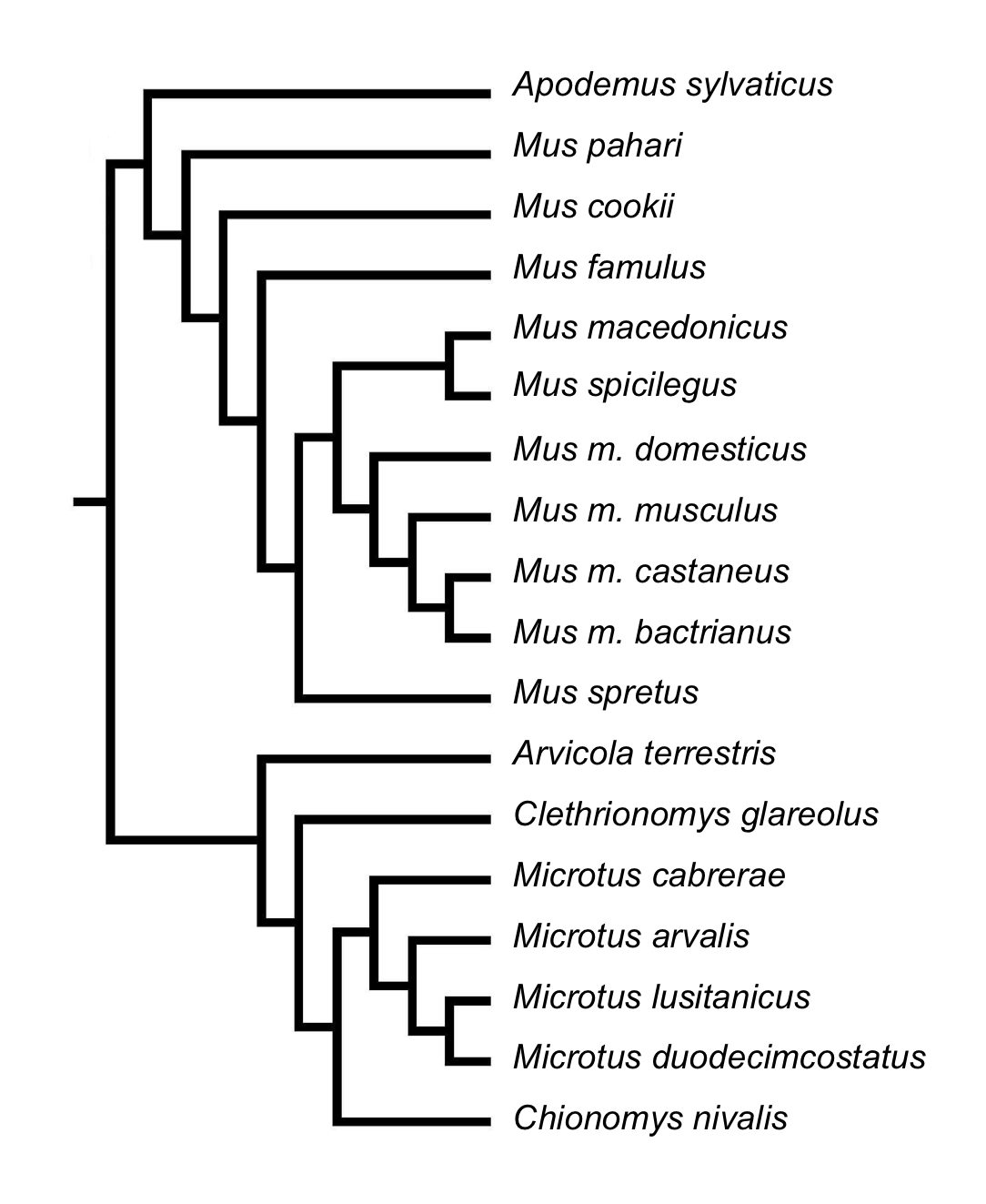

Supplement: Figure S2 — Reconstructed phylogenetic tree of the muroid species used in this study. The tree was constructed based on the literature and on the analysis of cytochrome b sequences Details are given in the Materials and Methods section. (TIF) [file pone.0018173.s002.tif]
